# Sustainable Lignocellulosic Composites Derived from Recycled Paper and Cardboard for Building Applications

**DOI:** 10.3390/polym18131623

**Published:** 2026-06-30

**Authors:** Mohammad Hassan Mazaherifar, Luminița-Maria Brenci, Maria Cristina Timar, Octavia Zeleniuc, Maria Violeta Guiman, Camelia Coșereanu

**Affiliations:** 1 Faculty of Furniture Design and Wood Engineering, Transylvania University of Brasov, B-dul Eroilor nr. 29, 500036 Brasov, Romania; mohammad.mazaherifar@unitbv.ro (M.H.M.); cristinatimar@unitbv.ro (M.C.T.); zoctavia@unitbv.ro (O.Z.); cboieriu@unitbv.ro (C.C.); 2 Faculty of Mechanical Engineering, Transilvania University of Brasov, B-dul Eroilor nr. 29, 500036 Brasov, Romania; violeta.guiman@unitbv.ro

**Keywords:** recycled paper and cardboard, waste valorization, sandwich composite, sound absorption, thermal insulation, sustainable materials

## Abstract

The valorization of post-consumer waste materials is an important strategy for reducing environmental impact and supporting circular material use. In this study, lightweight sandwich composites were developed using recycled paper and cardboard as core materials, producing sustainable panels for thermal and acoustic insulation. Core panels were manufactured from 100% paper, 100% cardboard, and a 50–50% paper–cardboard mixture. Environmentally friendly foaming agents were added to increase porosity and reduce density. The cores were subsequently combined with 3 mm medium-density fiberboard (MDF), 1 mm oak veneer, and date palm midrib fibers to provide different surface characteristics. The resulting sandwich composites were evaluated through standardized measurements of thermal conductivity and sound absorption coefficients. Microstructural characteristics were investigated using stereomicroscopy and scanning electron microscopy coupled with energy-dispersive X-ray spectroscopy (SEM–EDX). The results indicate that both the core composition and the type of face layer influence their performance. Whilst composites with cardboard-rich cores had higher porosity and better thermal insulation, introducing perforations and increasing the panel thickness improved sound absorption. The findings demonstrate that recycled paper and cardboard can be effectively used as sustainable raw materials to produce lightweight sandwich composites, tested at material scale, for non-structural interior insulation/acoustic panels.

## 1. Introduction

The growing demand for sustainable materials has intensified research into valorizing post-consumer waste within a circular economy framework. Paper and cardboard account for a significant fraction of municipal solid waste worldwide, and although recycling systems exist, a substantial portion still ends up in landfills or is used in low-value applications. Transforming these lignocellulosic residues into value-added materials offers an environmentally beneficial pathway for reducing waste accumulation while decreasing the demand for virgin raw materials. In this context, lightweight composite panels produced from recycled paper-based materials have attracted increasing interest for building and interior applications due to their low density, renewable origin, and potential for thermal and acoustic insulation. The lightweight panels can be designed as sandwich structures with a low-density core material, providing effective insulation and outer faces that confer rigidity and durability [1]. Core materials could include rigid foams such as expanded polystyrene, polyurethane, or phenolic materials [2,3,4]; fibrous insulation materials such as mineral wool or glass wool [5,6]; or cellular/honeycomb structures [7,8], each offering distinct advantages for specific applications. Foams are generally characterized by lower thermal conductivity, typically ranging from 0.018 W/m·K to 0.040 W/m·K, making them highly effective at minimizing heat transfer [9]. The foam cores usually exhibit relatively poor sound absorption properties, especially in the low-frequency range (100–800 Hz), depending on thickness and composition. This can be improved by introducing nano-fibers, reaching a value of 0.7 at frequencies in the range of 600–800 Hz [10]. Both glass wool and mineral wool perform well as insulation materials, with average thermal conductivities of 0.030–0.046 W/m·K [11,12]. When combined with sufficiently dense face sheets such as HDF or MDF, these sandwich panels can achieve sound transmission class (STC) ratings suitable for non-critical interior partition walls or building cladding applications. This could be due to the combination of a core material that absorbs sound and dense face sheets that block sound, creating an effective barrier against airborne noise. Nowadays, there is a growing interest in using lightweight panels made from recyclable materials. Waste materials, such as paper, cardboard, recycled textiles, and plastic, can be reused to create environmentally friendly materials with enhanced insulation properties, thereby contributing to waste reduction and resource conservation [13,14,15].

Globally, the recycling rate for paper products averages about 60%, with Europe leading at over 80% for packaging paper and cardboard [16]. Paper is a circular material that has one of the highest recycling rates; it can be recycled up to 25 times. Despite this, about 6 million tons of paper and cardboard packaging are sent to incineration or landfill every year [16]. The global paper recycling market is expected to reach approximately $57.7 billion by 2030, driven by the circular economy and concerns about environmental resource depletion [17]. One way to recycle is to incorporate processed waste paper and cardboard into various products, such as particleboard, fiber cement composites for ceilings and walls, wall insulation, and roofing [18,19,20,21]. Furthermore, recent studies have focused on using a range of waste materials, including paper and cardboard, to produce lightweight mycelium-based composites with high potential for thermal insulation and acoustic absorption [22,23,24,25,26,27]. The building sector is expected to develop new eco-lightweight panels that are characterized by high material and energy consumption and by immense pollution from CO_2_ emissions and the generation of residues. In the European Union, buildings account for 40% of final energy consumption and 36% of total CO_2_ emissions [28]. Studies on the acoustic and thermal insulation performance of sandwich structures with a recycled cardboard or paper core and a wood-based composite overlay are limited in the literature. Studies on the optimal acoustic performance of different cardboard-based panels and paper tubes [29] were conducted using computer modeling software. Sandwich structures comprising two cardboard honeycomb panels filled with cellulose fiber and covered with a perforated 2 mm kraft paper sheet achieved a higher sound absorption coefficient than a commercial gypsum panel. The thermal performance was evaluated by [30] for paper honeycomb structures with Formica sheets as an overlay, with a sandwich density of about 470 kg/m^3^, yielding a thermal conductivity coefficient of 0.11 W/m·K. Recycled cardboard and paper can be effective thermal insulators in sandwich panels, with thermal conductivities ranging from 0.034 W/m·K to 0.069 W/m·K, depending on the specific material composition and structure [31,32,33,34]. Composites made of 60% cardboard reinforced with 40% date palm fibers, with densities between 226,6 kg/m^3^ and 312.8 kg/m^3^, following a long drying process using a combination of oven-dry and air-dry methods [34], recorded thermal conductivity values ranging from 0.074 to 0.081 W/mK. The same proportion, but with different vegetable fibers, was used for composites, yielding densities in the range of 278.6–343.8 kg/m^3^, thermal conductivity values between 0.072 and 0.10 W/mK, and sound absorption coefficients between 0.4 and 0.8 [32]. Hemp shiv mixed with cardboard fiber [35], activated by citric acid and coated with gum arabic, was dried in an oven at 170 °C for 24 h, resulting in a porous composite with a thermal conductivity value between 0.02 W/mK and 0.03 W/mK and a sound absorption coefficient in the range of 0.85–0.95 for frequencies less than 2000 Hz. Recycled cardboard and corn starch as a binder, following a mixing process and an air-drying method, resulted in composites with thermal conductivity values between 0.066 and 0.092 W/mK [33]. Beyond concerns about using cellulose fibers for thermal and acoustic applications, recent studies propose their use in structural applications [36], which require mechanical strength comparable to that of commercial boards like OSB.

Although recycled paper and cardboard have been investigated as raw materials for new composites, limited information is available on the development of lightweight porous structures from their fibers. The novelty of this work lies in the development of such porous cores for sandwich composite panels via a foaming-assisted process that enables controlled porosity enhancement using various foaming additives, and an oven-drying method that allows a shorter curing time than reported in the literature for oven-drying, air-drying, and their combination [32,33,34,35]. The objective of this work was to investigate the relationship between the generated porous structure and functional performance through combined thermal, acoustic, and optical microscopy, as well as SEM–EDX analyses. The designed composites provide thermal and acoustic properties while facilitating the valorization of post-consumer waste materials, such as paper and cardboard. Enhancing their aesthetic and functional value by integrating overlays such as MDF, oak veneer, and date palm fibers was also investigated. Considering their lightweight structure, acoustic and thermal insulation capabilities, and customizable surface finishes, these sandwich composites show potential for commercial applications in sustainable interior building systems, including acoustic wall/ceiling panels, decorative insulation boards, partitions, and other non-structural architectural elements. Therefore, this research aims to address the existing gap by developing lightweight porous sandwich composites from paper and cardboard waste, optimizing their structure–performance relationship, and evaluating their potential as sustainable alternatives for non-structural interior applications through comprehensive thermal, acoustic, and microstructural characterization. Further research addressing mechanical performance, durability, and water affinity is required to overcome the current limitations and improve the applicability of these materials.

## 2. Materials and Methods

### 2.1. Materials and Composite Manufacturing

**Figure 1:**
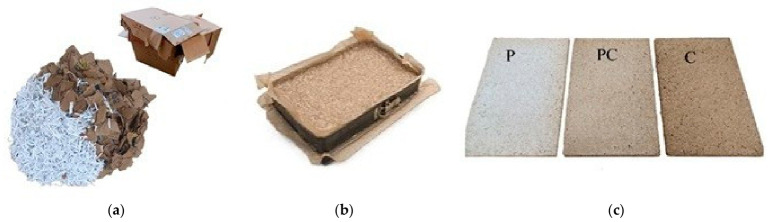
Core composite preparation: (**a**) Paper and cardboard waste; (**b**) mixture poured into the mold; (**c**) sized core panels, where P means 100% paper, PC means 50% paper/50% cardboard, and C means 100% cardboard.

The cores of the sandwich panels were manufactured from shredded office paper and fragmented cardboard packaging, as shown in Figure 1a, using three formulations: 100% paper, 100% cardboard, and a 50–50% paper–cardboard mixture. Water, sodium bicarbonate, and yeast were incorporated as foaming agents to enhance porosity and reduce the sample density.

The mixture composition was 15 wt.% solid material (paper, cardboard, or their mixture), 65 wt.% water, 12 wt.% sodium bicarbonate, and 8 wt.% yeast. The suspension was homogenized at 9000 rpm for 1 min, poured into a 350 × 270 × 30 mm mold lined with baking paper, as shown in Figure 1b, and cured at 150 °C for 15 h. In the next step, the specimens were cooled, then conditioned for 7 days at 20 °C and 65% relative humidity, and finally trimmed to 320 × 250 × 12 mm, as illustrated in Figure 1c. The final average density of the resulting composites was 220 kg/m^3^ for P-core, 161 kg/m^3^ for C-core, and 216 kg/m^3^ for PC-core. The porosity of the cores was measured using the Gas Pycnometer AccuPyc III 1350 (Micromeritics Instrument Corp., Norcross, GA, USA), with recorded values of 86% for P-cores, 90% for C-cores, and 87% for PC-cores.

Sodium bicarbonate acts as a chemical foaming agent, whilst the addition of yeast contributes to gas generation through fermentation, both releasing carbon dioxide (CO_2_), which becomes entrapped within the fiber-based matrix. The generated gas creates internal voids, increasing the porosity of the composite structure. At high temperature (150 °C), this porous morphology is retained, leading to a lower effective density. The proportions of sodium bicarbonate and yeast were selected based on preliminary experimental trials to balance pore formation and structural integrity. Excessive foaming agent content resulted in insufficient bonding between fibers and reduced mechanical stability, whereas lower amounts led to limited pore development.

**Figure 2:**
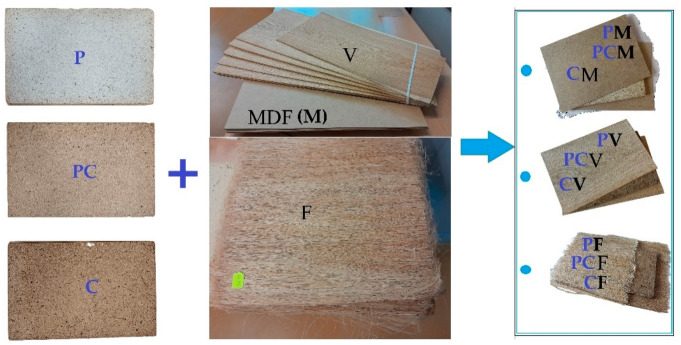
Cores (P for paper, C for cardboard, PC for paper and cardboard mixture), face layers (M for MDF faces, V for veneer faces, and F for date palm fiber faces), and sandwich panel formation (according to Table 1).

Three types of face layers were used to fabricate the sandwich composites with the cores shown in Figure 1c: 3 mm medium-density fiberboard (MDF), 1 mm oak veneer (V), and sheets of date palm midrib fibers (F) prepared as described by [37]. The face layers (Figure 2) were bonded to the cores using Jowacol 103.05 polyvinyl acetate adhesive D3 in an Italpresse GL6 hot press (Italpresse SPA, Bagnatica, Italy) at 1 N/mm^2^ and 50 °C for 20 min.

Adhesive consumption was optimized based on preliminary trials, with application rates of 500 g/m^2^ for MDF, 600 g/m^2^ for veneer, and 800 g/m^2^ for fiber sheets. The coding of the sandwich panels, their densities, and the moisture content of the cores and faces are presented in Table 1. Three replicates of each sandwich panel were manufactured, and the test specimens were subsequently cut. The moisture contents of the cores and faces, together with the densities presented in Table 1, were determined in accordance with European standards [38,39].

### 2.2. Thermal Insulation Performance of the Samples

The thermal conductivity coefficient (λ) of the composite panels was determined using a heat flow meter (HFM 436 Lambda, Netzsch, Selb, Germany), following the procedures outlined in [40,41]. The measurement principle is based on measuring the heat flux through the specimen positioned between a hot plate (with T2 temperature) and a cold plate (with T1 temperature) under steady-state conditions. The resulting temperature gradient across the sample was continuously monitored, and the λ value was automatically calculated by the system software using Fourier’s law of heat conduction. Instrument calibration was conducted before testing to ensure measurement accuracy, including the mean temperature (Tm) and the temperature difference (∆T). During the experiments, the hot plate was kept constant at 20 °C, while the cold plate temperature was varied from −10 °C to 15 °C in 5 °C intervals. Three replicates, each measuring 300 mm × 250 mm × 30 mm, were tested. For each sample, six measurements were performed under different temperature differences (ΔT) and mean temperatures (Tm), as presented in Table 2. The final λ-value was calculated as the average of the six measurements.

### 2.3. Acoustic Performance Testing of the Samples

The acoustic absorption coefficient was investigated with a Brüel & Kjær impedance tube system (Type 4206, Nærum, Denmark). For each formulation, disk-shaped specimens (29 mm in diameter) were cut by using a router from both the face layers of the sandwich composite and the core layers; one of these specimens incorporated seven circular perforations, each 3 mm in diameter (Table 3). The calculated perforation ratio is 7.5%.

The absorption coefficient was determined within the 50 Hz–6400 Hz frequency range using the transfer function method with two microphones, in compliance with standards [42,43]. The method is based on the separation of incident and reflected plane waves and uses acoustic pressure measurements to calculate the material’s acoustic properties via a two-microphone transfer function. Measurements were recorded and processed using the Pulse software package—PULSE LabShop Version 12.1.0 (Brüel & Kjær). A total of 51 specimen configurations were evaluated, derived from different combinations of surface layers and core materials (Table 2), including 24 single-layer and 27 double-layer configurations. Three replicates of each type of sandwich composite were measured, and the mean values were recorded to calculate the Noise Reduction Coefficient (NRC) and the Sound Absorption Average (SAA) values. NRC was calculated as the mean value of absorption coefficients at 250, 500, 1000, and 2000 Hz, whilst SAA was calculated as the average of absorption coefficients across 12 one-third-octave bands from 200 Hz to 2500 Hz.

The design variations were intended to examine the effects of material type, overall thickness, and perforation on the acoustic behavior of the sandwich composites. During the test, the samples were positioned in the Kundt tube so that the incident sound waves first encountered the overlaid surface, followed by the additional core layers.

## 3. Results and Discussion

### 3.1. Thermal Insulation Characterization of the Sandwich Composites

**Figure 3:**
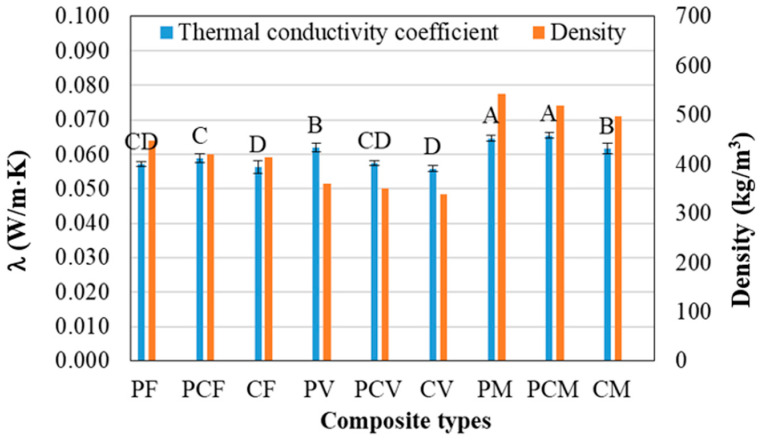
Thermal conductivity coefficient (λ) vs. density of the panels (acronyms as shown in Table 1).

The recorded thermal conductivity coefficient and density values of the developed composites are presented in Figure 3. Statistical analysis was conducted to evaluate differences among the composite groups. Standard deviations were calculated using Microsoft Excel with a 95% confidence level and a significance threshold of α = 0.05 (*p* < 0.05). The significance of differences between groups was determined using a two-sample *t*-test in Minitab software (version 19.2020.1). The statistical grouping in Figure 3 shows that PM and PCM composites overlaid with MDF exhibited the highest densities, 520 kg/m^3^ and 544 kg/m^3^, respectively, and were classified in Group A, with significantly higher thermal conductivity values (around 0.065 W/m·K). Similar values were also reported for composites made of cardboard mixed with cornstarch as a binder, with lower densities of around 450 kg/m^3^ in a previous study [33].

In contrast, panels made of 100% cardboard and overlaid with date palm fibers (CF) and veneer (CV), with lower densities of 415 kg/m^3^ and 350 kg/m^3^, respectively, were classified in Group D and recorded the lowest λ values (≈0.056 W/m·K). The other sandwich composites, such as PF, PCF, PV, PCV, and CM, were distributed among Groups B, C, and CD, showing moderate thermal conductivity values. Their overlap in statistical groupings indicates that, in addition to density, other factors, such as composition, fiber morphology, and interfacial bonding characteristics, influence heat transfer. For instance, PV exhibited a lower density but still showed conductivity comparable to that of denser composites, suggesting that material distribution and bonding efficiency also contribute significantly to the observed values. Similarly, Refs. [32,34] reported thermal conductivities comparable to or even higher than those of composites comprising 60% cardboard waste and 40% natural fibers. These materials exhibited lower densities, ranging from 220 kg/m^3^ to 320 kg/m^3^. The similarity in conductivity despite reduced density suggests that fiber type and its distribution may also play a decisive role in thermal performance, beyond the effect of apparent density alone. Another example is the work of researchers [31], who developed sandwich structures with a density of about 470 kg/m^3^ and achieved a high thermal conductivity of 0.11 W/m·K. In highly porous bio-composites such as mycelium-based composites [24], low density ranging from 107 kg/m^3^ to 227 kg/m^3^ and high porosity are key factors in maintaining a thermal conductivity coefficient in the range 0.043 W/m·K–0.056 W/m·K. The above comparisons reinforce the idea that, while density is a dominant factor in the thermal insulation of the composites, their structural characteristics, such as porosity and binder–fiber adhesion, also modulate thermal behavior, accounting for the intermediate performance observed in PF, PCF, PV, PCV, and CM composites. From an application perspective, the developed sandwich composites are promising candidates for thermal insulation in sustainable building materials, with thermal conductivity coefficients ranging from 0.055 to 0.065 W/m·K. Compared with conventional insulation materials [2,3,4,5,6], which possess highly controlled pore structures, the cores of the developed sandwich composites contain heterogeneous fibrous networks with interconnected pores, and their surface layers are denser solid materials, which increases the overall thermal conductivity. These factors explain the observed values, while the panels still demonstrate promising insulation performance, considering their recycled and renewable material composition. Overall, these results emphasize the importance of tailoring density, materials, and internal structure when designing sustainable composites for thermal insulation and also highlight the development of environmentally friendly materials.

### 3.2. Sound Absorption Characterization of the Samples

**Figure 4:**
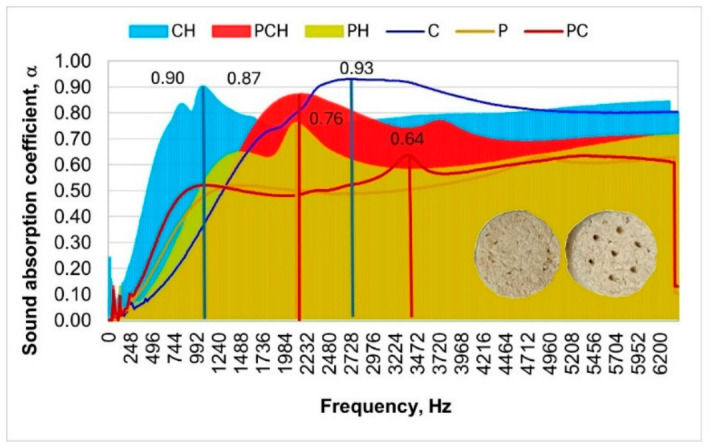
Acoustic performance of the core materials used in the structure of sandwich composites (acronyms as shown in Table 1 and Table 3).

The sound absorption coefficient (α) values for the paper- and cardboard-based materials used as cores in the developed sandwich composites are shown in Figure 4. The cardboard-based cores (C and CH) outperform the other materials in terms of maximum absorption capacity, reaching 0.93 at approximately 2800 Hz and 0.90 at approximately 1000 Hz, respectively. The C core also maintains the highest steady absorption in the upper register, staying above 0.80. The beneficial contribution of cardboard to sound absorption is evident in the PCH results, which show a sound absorption peak of 0.87 at approximately 2000 Hz compared to the moderate values achieved by paper-based cores (P and PH). For all materials, the contribution of the holes is seen in the narrowing of the frequency range over which sound absorption peaks occur.

**Figure 5:**
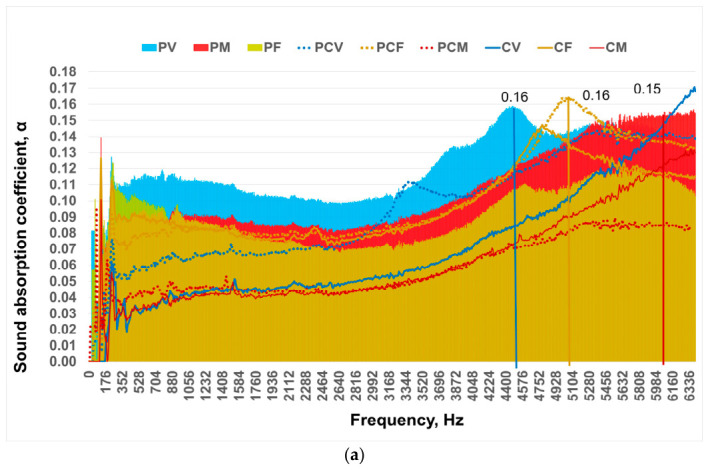

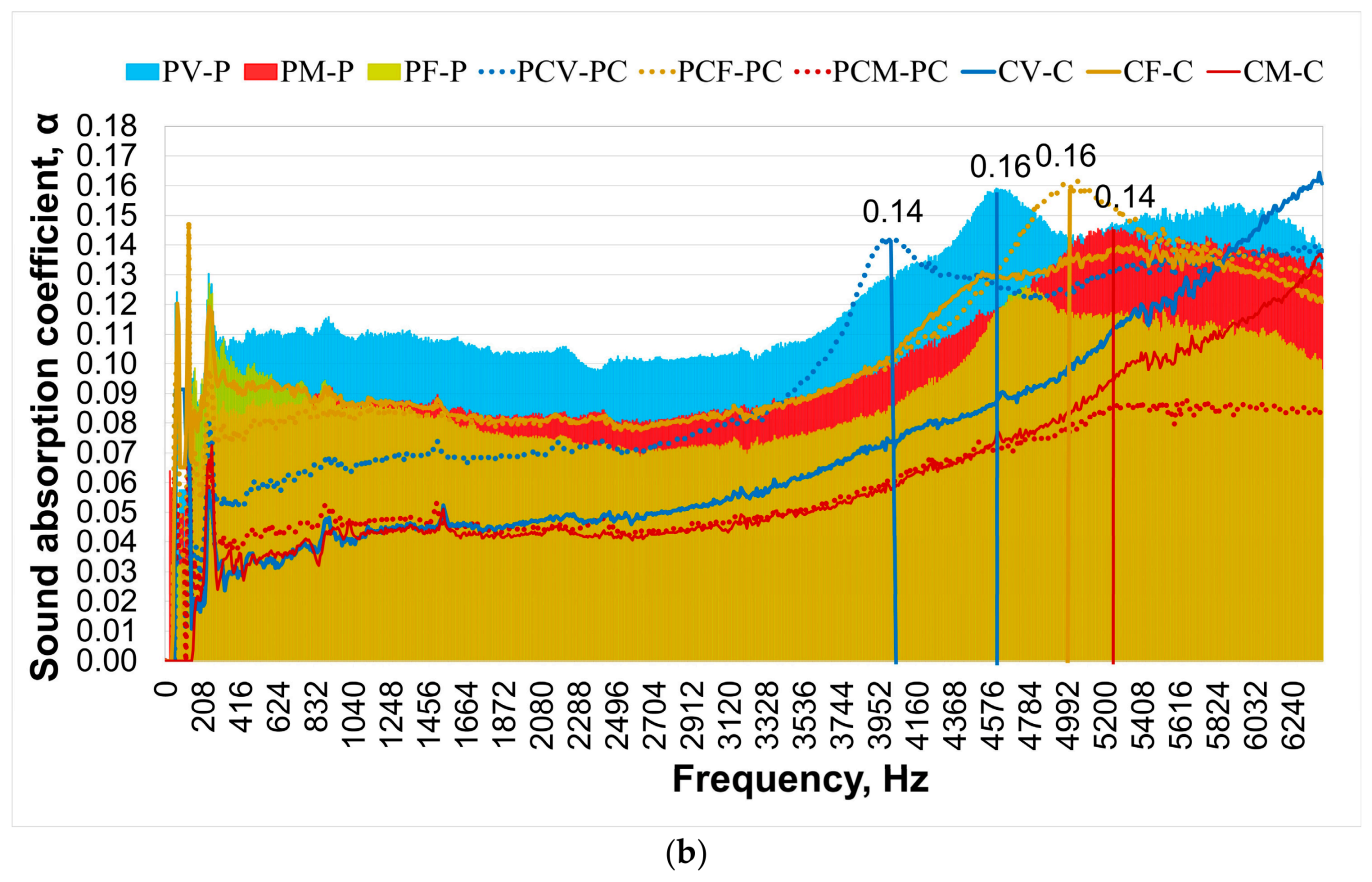
Acoustic performance of the overlaid materials: (**a**) single-layer configurations with overlaid materials; (**b**) double-layer configurations, adding unperforated cores (acronyms as shown in Table 1 and Table 3).

When overlaying the unperforated cores (C, PC, and P), regardless of the overlay material (MDF, veneer, or date palm fiber sheets), the sound absorption coefficient recorded very low values, no more than 0.16, showing that the sound is mostly reflected by these materials, as shown in Figure 5a. The same low performance is maintained when an unperforated core is added to the overlaid material, as seen in Figure 5b.

**Figure 6:**
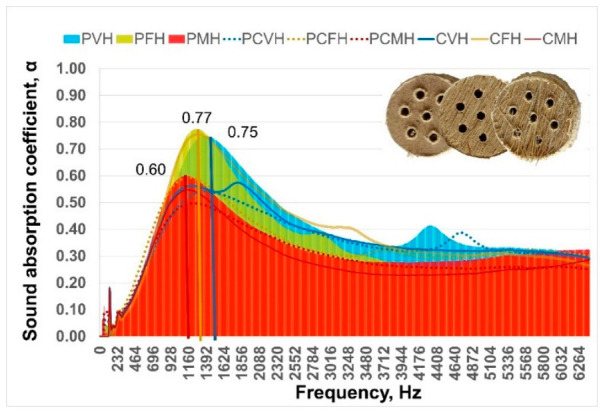
Sound absorption coefficient values of the perforated overlaid composites (acronyms as shown in Table 3).

When the overlaid composites were perforated, higher sound absorption coefficients were recorded (Figure 6). The results presented in Figure 6 demonstrate that perforations enhance the acoustic absorption, transforming the weakly absorbing panels (α ≈ 0.1–0.2) into effective sound absorbers. Among the perforated samples, the PFH, PVH, and CFH composites exhibited the highest performance, with peaks above 0.75. The similarity in their absorption levels suggests that both paper and cardboard cores, when covered with date palm fiber layers, can achieve comparable efficiency in the frequency band crucial to speech clarity [44].

PFH composite peaked slightly earlier (1192–1344 Hz), while CFH and PVH extended toward higher frequencies (up to 1300 Hz). As noted by [28], large perforations result in higher peak frequencies, and low porosity and density enable deeper, more efficient sound-wave absorption at higher frequencies. Furthermore, more porous surface samples have higher sound absorption coefficients [23], as observed in this study for composites with fiber and oak veneer surface layers.

Intermediate performance was observed for PMH and PCFH (α ≈ 0.60), whose broader but lower absorption indicates that MDF and mixed cardboard–paper covered with a fiber overlay introduce additional damping but do not maximize the air-resonance effects in the perforations. The CVH, CMH, and PCVH composites exhibited moderate absorption (α ≈ 0.54–0.55) over a wider frequency range, suggesting that these layer configurations distribute energy dissipation more evenly but reduce peak efficiency. The PCMH composite performed the worst (α = 0.50), likely due to the MDF exterior layer, in which the stiffer face layer reduces porosity-driven absorption mechanisms.

**Figure 7:**
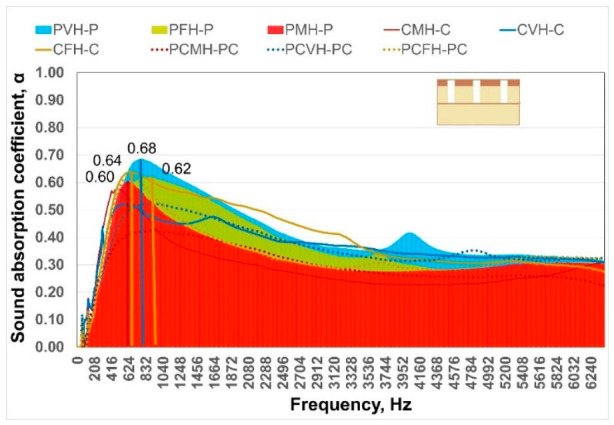
Sound absorption coefficient values of the double-layer sandwich-type composite samples composed of a perforated overlaid composite and one layer of unperforated core (acronyms as shown in Table 3).

As shown in Figure 7, increasing the thickness of paper-based composites by combining a perforated facing with a non-perforated core reduced the peak sound absorption coefficient while shifting the effective absorption toward lower frequencies.

This response reflects Helmholtz-type resonance and mass–spring behavior, in which increased thickness lowers the effective acoustic absorption and the resonance frequency [28]. The accompanying rise in damping broadens the absorption band, thereby enhancing low-frequency performance at the expense of peak amplitude.

**Figure 8:**
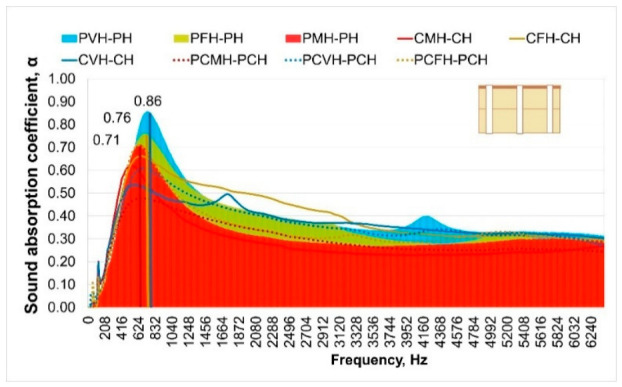
Sound absorption coefficient values of the double-layer sandwich-type composite samples composed of a perforated overlaid composite and one layer of perforated core (acronyms as shown in Table 3).

Adding a second perforated core (Figure 8) further enhanced performance: peaks were observed at α = 0.86 (720–752 Hz) for PVH-PH, α = 0.76 (664–736 Hz) for PFH-PH, and α = 0.71 (600–680 Hz) for PMH-PH. These results demonstrated that increasing sample thickness reduced the absorption peak frequency and increased its maximum value—but only when the additional layer was perforated, thereby strengthening air resonance within the holes.

As with the single-layer samples, the highest α-values were obtained for those covered with veneer and date palm fibers, while MDF overlays showed the lowest performance. Since frequencies below 1000 Hz correspond to fundamental speech sounds, guitar tones, and piano mid-range notes, absorber thickness can be tuned to target specific frequency ranges.

Overall, the results highlight four main factors controlling acoustic behavior, namely (i) stiffness and porosity of the face layer, which determine the frequency position of absorption peaks; (ii) core structure, which governs resonance strength; (iii) thickness and damping properties of second layers, which are crucial for enhancing low-frequency absorption; and (iv) the presence of perforations, which favors Helmholtz resonance and has a positive effect on the acoustic absorption.

The Noise Reduction Coefficient (NRC) and Sound Absorption Average (SAA) are presented in Table 4, calculated for three samples each over the 200–2000 Hz frequency range, which corresponds to the range most perceptible to humans. These results place the composites in the moderate-absorption category, well above rigid, non-absorptive materials such as MDF or veneer, which typically have NRC values of ≤0.15. The most effective configurations combined perforated cores with lightweight, porous face layers such as date palm fibers or oak veneer, confirming the importance of resonance effects and surface porosity in enhancing acoustic response. Importantly, these composites were produced entirely from recycled or bio-based raw materials, making them lightweight, sustainable alternatives to synthetic absorbers. Their combination of moderate sound absorption, aesthetic versatility, and environmental benefits suggests strong potential for use in interior applications where sustainability and functional acoustic treatment are equally important.

### 3.3. Microscopic Morphology of the Samples

**Figure 9:**
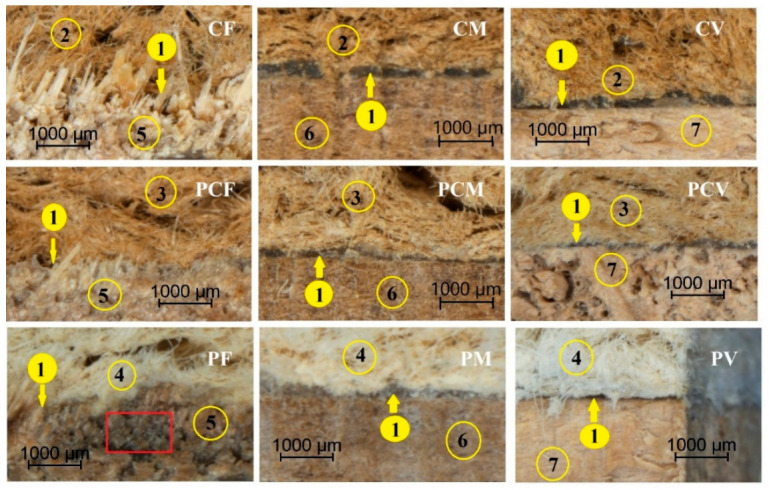
Bonding areas between cores and overlays of the developed sandwich composites at 22.5× magnification (1—bonding area; 2—cardboard core; 3—mixed paper–cardboard core; 4—paper core; 5—date palm fiber overlay; 6—MDF overlay; 7—veneer overlay) observed under an optical stereomicroscope (acronyms as shown in Table 1).

The microscopic images in Figure 9 reveal distinct differences in adhesion at the core–overlay interfaces of the sandwich composites. In the cardboard-based panels (CF, CM, CV), there is no visible adhesion line between the cardboard core and date palm fibers overlay (CF), which could be explained by a deeper adhesive penetration into the fibrous structure, whilst MDF (CM) and veneer (CV) interfaces appear as irregular lines with random distribution of the adhesive layer. In addition, multiple voids can be observed in the cardboard core structure (marked with 2), indicating a low-density, porous material, a conclusion supported by the measured porosity of 90%.

For the mixed paper–cardboard cores (PCF, PCM, PCV), the bonding areas appear more compact and thinner, but the core (marked with 3) presents densified areas of material where white fibers are more numerous, indicating the presence of paper fibers, which positively contribute to the higher density of this type of core (216 kg/m^3^, compared to 161 kg/m^3^ for only cardboard-based C-core).

Paper-based panels (PF, PM, PV) generally exhibited thinner bonding layers and localized adhesive penetration in the fiber region, whereas MDF overlays (PM) provided a relatively uniform interface. Veneer overlays (PV) exhibited a more uniform, but narrower, bonding line, indicating more superficial adhesion than fiber overlays. All images of the paper-based samples show a more compact paper core (marked with 4), indicating a less porous structure (measured porosity of 86% versus 90% for the C-core). Overall, the images suggest that: (i) date palm fiber overlays promoted better adhesive penetration; (ii) MDF and veneer overlays formed more uniform and continuous bonding layers but showed non-uniform adhesion with the cardboard core (CM); and (iii) high porosity and low fiber inter-connection for cardboard-based cores explain the better thermal insulation capacity.

### 3.4. SEM–EDX Investigation

**Figure 10:**
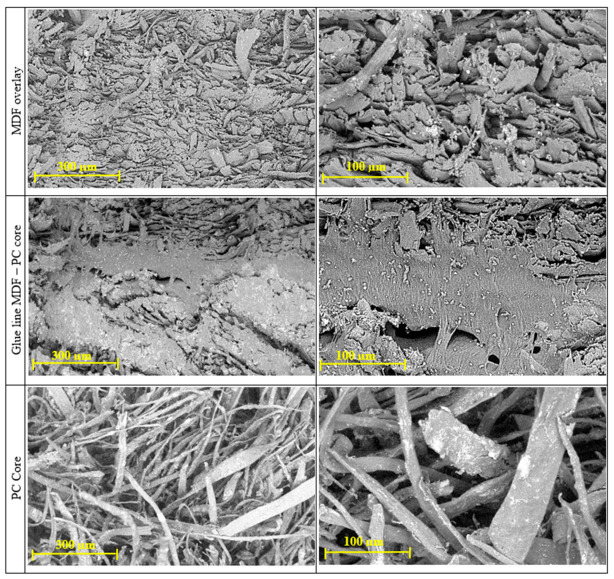
SEM images of PCM composite showing the fibrous structure and different porosity of the MDF overlay (**top**) and the PC core (**bottom**), alongside the bonding area with the glue line and the two interfaces (**middle**); 500× magnification on the left side and 1500× on the right side.

A selection of scanning electron microscopy (SEM) images at 500× (left) and 1500× (right) magnification of the MDF-overlaid paper–cardboard composite panels (PCM), highlighting comparatively relevant structural features of the MDF overlay compared to the PC core, alongside the bonding area with the glue line and adhesive-penetrated areas at the interface level, is presented in Figure 10.

Both the MDF overlay and the PC core are fibrous materials with a porous, heterogeneous structure comprising areas of varying compactness; however, their overall porosities differ, with the PC core having a higher porosity than the MDF overlay.

The fibers in the PC composite appear longer and more individual, resembling long ribbon-like cells characteristic of resinous wood pulp [45], whereas those in the MDF are compacted and bound together by the adhesive used in the manufacturing process. A glue line with variable thickness is clearly visible, with evident adhesive penetration and anchorage in both adherents at the interfaces. Additionally, in the more porous PC core, adhesive impregnation into the substrate is evident.

**Figure 11:**
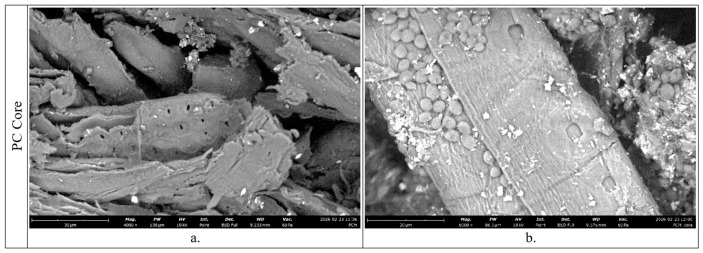
Structural details revealed by SEM: (**a**) wood fibers with visible bordered pits (MDF) at 4000× magnification; (**b**) fiber with bordered pits and agglomeration of yeast granules (PC core) at 6000× magnification.

At higher magnifications, details of the wood fiber’s anatomical structure, such as bordered pits and crushed cells in the MDF layer, or individual fibers with bordered pits and agglomerations of yeast in the paper–cardboard core, are visible via SEM (Figure 11). The SEM image in Figure 11b, showing yeast cells fixed to the surface of a cellulosic fiber, suggests an affinity of the yeast cells for cellulose fibers. A quite similar micro-morphological pattern of interaction between yeast (*Saccharomyces cerevisiae*) and cellulose fibers was previously reported by [46] in a study exploring the potential of a synergic bio-absorbent based on yeast fixed on cellulose fibers, and by [47] in a study of yeast strains. The physical interactions of the polar groups, amino (-NH2) and hydroxyl (-OH), in yeast with the hydroxyl groups in cellulose are a possible explanation.

**Figure 12:**
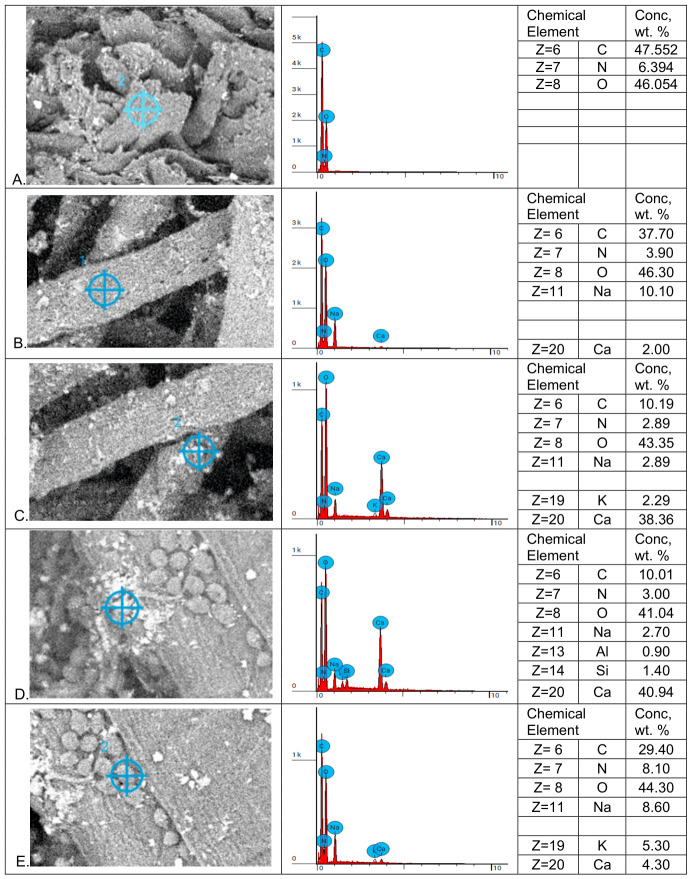
Results of the SEM–EDX analysis of the PC core of the PCM composite at different points (marked with a cross in blue circles).

Energy-Dispersive X-ray Spectroscopy (EDX) analysis was performed at different points/areas within the SEM images, identifying specific features by distinctive shapes or chemical contrast (e.g., whitish areas). Some results obtained in the investigation of the MDF overlay and the PC core of the PCM sandwich composite are presented in Figure 12. For MDF, EDX analysis at the fiber surface reveals the presence of C (~47.5%), O (~46.0%), and N (~6.4%), the latter attributed to the urea–formaldehyde adhesive (Figure 12A). A similar analysis carried out on the fibrous material of the foamed PC core reveals a different composition, with less C (~37.7%), a similar content of O (~46.3%), less N (~3.9%), and the presence of Na (~10.10%) and some Ca (2.0%).

Considering the materials and preparation methods used in this research to obtain the core, it is reasonable to assume that nitrogen derives from the protein content of the yeast cells, sodium from the sodium bicarbonate used as a foaming agent, and calcium carbonate from a common filler used in paper manufacturing. Other chemical elements, such as silicon (Si), aluminum (Al), magnesium (Mg), sulfur (S), potassium (K), zinc (Zn), and titanium (Ti), were frequently detected in paper and cardboard due to their use as fillers or coatings [46,48].

The smaller or larger granular white deposits on the PC fibers (Figure 12B–D) are characterized by higher Ca content (30–40%) and small amounts of K, Al, and Si (1–5%). Areas with such a granular, whitish, Ca-rich appearance were also detected on the cut surface of the MDF overlay, and we assume that this is more likely contamination from the PC core during sample preparation by cutting than a component of the MDF. Finally, when the analysis spot was placed on a yeast cell (Figure 12E), a higher N content (~8.10%) was detected due to the yeast’s composition (C, O, N). Similar results from SEM–EDX have been reported in the literature [46].

The microscopy and SEM observations revealed differences in fiber arrangement, surface morphology, and visible pore structure among the developed composites. These observations suggest variations in material compactness and interface characteristics; however, quantitative measurements of adhesive penetration and interfacial bonding were not performed. The EDX analysis identified the presence of elements such as C, O, Na, Ca, and N, which may be associated with the raw materials and processing additives.

### 3.5. Techno-Economic Considerations and Future Research Perspectives

As a result of the present research, the developed paper-and-cardboard sandwich composites may be alternatives for lightweight interior insulation and acoustic applications; however, several techno-economic aspects require further investigation before large-scale implementation. The use of post-consumer waste materials offers advantages in raw material availability, waste reduction, and circular material utilization. Nevertheless, challenges remain in waste collection, sorting, cleaning, fiber preparation, and the consistency of material quality, which may affect production costs and process reliability.

From a manufacturing perspective, the proposed foaming-assisted process and drying method offer opportunities to reduce material density and improve functional performance; however, optimizing energy consumption, processing time, and equipment requirements is necessary to assess industrial feasibility.

For commercial applications, additional surface and structural treatments of the developed composites may be considered in further research to enhance durability without affecting thermal or acoustic performance. Water-repellent products applied to the surface layers and used to seal the edges, along with flame-retardant and anti-fungal additives incorporated into the core, could improve resistance to moisture, fire, and mold, without significantly affecting aesthetic characteristics. On the other hand, the sandwich configuration improves mechanical performance, as the MDF, oak veneer, and date palm fiber face layers reinforce the recycled paper/cardboard core; however, further improvement can be achieved by incorporating functional nanoparticles into the core, thereby enhancing its durability.

## 4. Conclusions

This study demonstrated the feasibility of producing lightweight sandwich composites from recycled paper and cardboard waste using a foaming-assisted approach to enhance porosity and functional performance.

The thermal results showed that cardboard-core composites with oak veneer and date palm fiber overlays (CV and CF) achieved the lowest thermal conductivity values (λ = 0.056 W/m·K), while MDF-faced structures exhibited higher values (≈0.065 W/m·K).

Acoustic evaluation revealed that perforated porous composites with lightweight overlays (PFH and PVH) reached a maximum sound absorption coefficient of approximately 0.76 at frequencies above 1200 Hz, while the double-layer PVH-PH configuration achieved a peak absorption coefficient of 0.86 within the 720–752 Hz range.

SEM–EDX analysis revealed that the MDF and PCM cores were non-homogeneous fibrous materials with different porosities and corresponding local variations in elemental composition.

## Figures and Tables

**Table 1:** Sandwich panel architecture and characteristics.

Panel Code	Core Panel Raw Material	Face Layers	Density	Moisture Content (%)	
			(kg/m^3^)	Core	Faces
PM	100% paper (P)	MDF (M)	544	6.6	6.7
PV	100% paper (P)	Veneer (V)	360	6.6	7.5
PF	100% paper (P)	Date palm fibers (F)	448	6.6	6.3
PCM	50% paper/50% cardboard (PC)	MDF (M)	520	8.5	6.7
PCV	50% paper/50% cardboard (PC)	Veneer (V)	350	8.5	7.5
PCF	50% paper/50% cardboard (PC)	Date palm fibers	420	8.5	6.3
CM	100% cardboard (C)	MDF (M)	497	9.5	6.7
CV	100% cardboard (C)	Veneer (V)	339	9.5	7.5
CF	100% cardboard (C)	Date palm fibers (F)	415	9.5	6.3

**Table 2:** The parameter sets for testing the λ-value.

Test No.	T1 of the Cold Plate	T2 of the Hot Plate	ΔT = T2 − T1	Tm = (T1 + T2)/2
1	−10	20	30	5
2	−5	20	25	7.5
3	0	20	20	10
4	5	20	15	12.5
5	10	20	10	15
6	15	20	5	17.5

**Table 3:** Sandwich panel coding and structure for sound absorption testing.

Recycled Materials for the Cores	Single-Layer Configurations and Their Codes			Double-Layer Configurations and Their Codes		
	Face Layers	Overlaid Composites and Their Codes 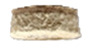	Additional Cores and Their Codes	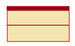 No Perforations	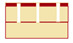 One Perforated Layer	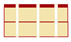 Two Perforated Layers
Paper (P)	MDF (M)	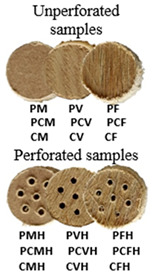	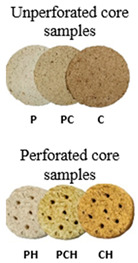	PM-P	PMH ^1^-P	PMH-PH
	Veneer (V)			PV-P	PVH-P	PVH-PH
	Fibers (F)			PF-P	PFH-P	PFH-PH
	
Paper and cardboard (PC)	MDF (M)			PCM-PC	PCMH-PC	PCMH-PCH
	Veneer (V)			PCV-PC	PCVH-PC	PCVH-PCH
	Fibers (F)			PCF-PC	PCFH-PC	PCFH-PCH
	
Cardboard (C)	MDF (M)			CM-C	CMH-C	CMH-CH
	Veneer (V)			CV-C	CVH-C	CVH-CH
	Fibers (F)			CF-C	CFH-C	CFH-CH
	

^1^ H denotes with a sample with holes (perforated sample).

**Table 4:** SAA and NRC of the composite samples ^1^.

100% Paper-Based Composites			50% Paper + 50% Cardboard-Based Composites			100% Cardboard-Based Composites			Layers
Type	SAA	NRC	Type	SAA	NRC	Type	SAA	NRC	
PFH	0.35 (0.005)	0.35 (0.015)	PCFH	0.33 (0.004)	0.34 (0.010)	CFH	0.36 (0.005)	0.36 (0.003)	1
PVH	0.34 (0.005)	0.34 (0.017)	PCVH	0.30 (0.004)	0.31 (0.004)	CVH	0.32 (0.004)	0.34 (0.007)	1
PMH	0.31 (0.003)	0.32 (0.013)	PCMH	0.29 (0.003)	0.29 (0.010)	CMH	0.29 (0.004)	0.29 (0.006)	1
PFH-P	0.43 (0.008)	0.44 (0.013)	PCFH-PC	0.41 (0.007)	0.42 (0.006)	CFH-C	0.48 (0.005)	0.49 (0.010)	2
PVH-P	0.45 (0.010)	0.46 (0.002)	PCVH-PC	0.40 (0.008)	0.40 (0.011)	CVH-C	0.42 (0.002)	0.42 (0.011)	2
PMH-P	0.40 (0.010)	0.41 (0.014)	PCMH-PC	0.35 (0.006)	0.35 (0.011)	CMH-C	0.39 (0.010)	0.40 (0.010)	2
PFH-PH	0.45 (0.007)	0.45 (0.015)	PCFH-PCH	0.42 (0.006)	0.43 (0.009)	CFH-CH	0.47 (0.006)	0.48 (0.013)	2
PVH-PH	0.44 (0.008)	0.44 (0.014)	PCVH-PCH	0.42 (0.006)	0.42 (0.008)	CVH-CH	0.42 (0.005)	0.42 (0.012)	2
PMH-PH	0.41 (0.007)	0.41 (0.013)	PCMH-PCH	0.36 (0.006)	0.37 (0.007)	CMH-CH	0.39 (0.004)	0.40 (0.006)	2

^1^ Acronyms are shown in Table 3.

## Data Availability

The original contributions presented in this study are included in the article. Further inquiries can be directed to the corresponding author.
